# Preparation of Metallized Pellets for Steelmaking by Hydrogen Cooling Reduction with Different Cooling Rates

**DOI:** 10.3390/ma17174362

**Published:** 2024-09-03

**Authors:** Guanwen Luo, Zhiwei Peng, Kangle Gao, Wanlong Fan, Ran Tian, Lingyun Yi, Mingjun Rao

**Affiliations:** School of Minerals Processing and Bioengineering, Central South University, Changsha 410083, China; 225601020@csu.edu.cn (G.L.); 8204223001@csu.edu.cn (K.G.); 225611071@csu.edu.cn (W.F.); 215601041@csu.edu.cn (R.T.); yilingyun@csu.edu.cn (L.Y.); mj.rao@csu.edu.cn (M.R.)

**Keywords:** hydrogen reduction, iron ore pellets, cooling rate, metallization degree, porosity, metallic iron particles

## Abstract

To utilize the sensible heat of hot roasted iron ore pellets with no CO_2_ emission in the production of metallized pellets for direct steelmaking, the pellets were reduced in H_2_ during their cooling process with variable cooling rates. When the cooling rate decreased from 5.2 °C/min to 2.0 °C/min, the total iron content, reduction degree, and iron metallization degree of the pellets increased continuously from 74.0 wt%, 52%, and 31.1% to 84.9 wt%, 93.4%, and 89.2%, respectively. However, the compressive strength of the pellets increased initially from 2100 N/p to 2436 N/p and then decreased considerably to 841 N/p. As the cooling rate decreased, more Fe_2_O_3_ was reduced to Fe with diminishing FeO and Fe_2_SiO_4_. The porosity of the pellets increased from 23.9% to 54.3%, with higher distribution uniformity of pores. The morphology of metallic iron particles also transited from a layered form to a spherical form and lastly to a porous reticular form. Meanwhile, the metallic iron particles in the pellets grew evidently with more uniform distributions. When the cooling rate was 3.7 °C/min, the resulting metallized pellets had the reduction degree of 74.2%, iron metallization degree of 66.9%, and the highest compressive strength of 2436 N/p, in association with the spherical morphology and relatively large size of metallic iron particles.

## 1. Introduction

With the continuous consumption of fossil fuels in the process of economic development, the global CO_2_ emissions have been kept increasing in the past decades [1]. Among carbon-intensive industries, the iron and steel industry is one of the biggest CO_2_ emitters [2]. China’s iron and steel industry is dominated by the “blast furnace-basic oxygen furnace” process, which has a huge demand for traditional fossil fuels such as coke and coal [2,3]. Although the CO_2_ emission from blast furnace ironmaking has been significantly reduced through technological innovation and the development of carbon dioxide capture technology, developing green ironmaking technology without the involvement of fossil fuels is still the main strategic direction for the industry [4,5].

The use of hydrogen for ironmaking has become essential to address the problems associated with the declining availability and quality of natural resources and to fulfill China’s imminent “dual carbon” target [6]. Currently, the main hydrogen ironmaking technologies include hydrogen-rich blast furnace ironmaking [7], hydrogen direct reduction (H-DR) [8,9,10], hydrogen plasma smelting reduction (HPSR) [11,12,13], and hydrogen flash ironmaking [14]. Hydrogen-rich blast furnace ironmaking has been pioneered in industrial production in Germany and Japan [15,16]. However, because coke acts as the structural framework of the blast furnace and cannot be completely replaced, hydrogen-rich blast furnace ironmaking is expected to reduce CO_2_ emission limitedly (up to 20%) [17]. H-DR was developed based on the shaft furnace direct reduction process. Midrex and HYL are two typical H-DR processes which use a mixed gas of H_2_ and CO for reduction, showing a huge CO_2_ reduction capacity [18,19]. HPSR and hydrogen flash ironmaking may use 100% H_2_ for smelting. In theory, they can achieve zero CO_2_ emission [5]. Nevertheless, they are far away from industrial applications.

For hydrogen reduction, it has a better reduction performance than carbothermic reduction at high temperatures associated with the smaller specific gravity and higher diffusivity of H_2_. After reduction, there are usually obvious changes in the physicochemical properties of the feed materials, usually iron ore pellets which are prepared by drying of green pellets produced via traditional pelletizing of iron concentrate with a small amount of binder, followed by high-temperature preheating and then roasting [20,21]. It was shown that the temperature and atmosphere during the reaction process had significant effects on the pore structure and swelling characteristics of the resulting metallized pellets or direct reduced iron (DRI). The changes in pore structure also affected the diffusivity of H_2_ inside the pellets and ultimately the reaction kinetics. It was found that nascent metallic iron particles would transform into abundant whiskers, which might cause serious sticking between the pellets that lowered the product quality and production efficiency [22].

To make use of sensible heat of hot iron ore pellets after high-temperature roasting, which could avoid cooling and reheating needed for traditional hydrogen reduction processes, this study explored the hydrogen reduction characteristics of the pellets during their cooling process, called the hydrogen cooling reduction (HCR) process. The effect of cooling rate on the reduction process and the properties of the resulting metallic pellets was evaluated in detail.

## 2. Materials and Methods

### 2.1. Materials

**Figure 1:**
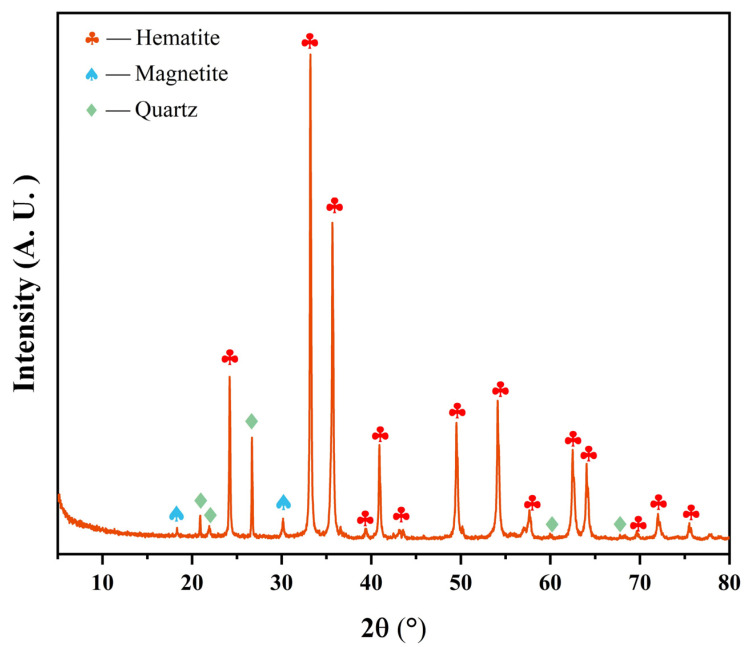
XRD pattern of iron ore pellets.

Table 1 shows the main chemical composition of iron ore pellets which were produced from iron concentrate in a conventional way after pelletizing, drying, preheating, and roasting successively. The total iron content (TFe) of the pellets was 64.00 wt% and those of SiO_2_, Al_2_O_3_, and CaO were 8.51 wt%, 0.42 wt%, and 0.18 wt%, respectively. The contents of harmful elements, such as P and S, were relatively low. Table 2 shows the basic physical properties of the pellets. They had a relatively uniform size and high compressive strength. Figure 1 shows the phase composition of the pellets. Its main mineral phase was hematite, with small amounts of magnetite and quartz.

**Figure 2:**
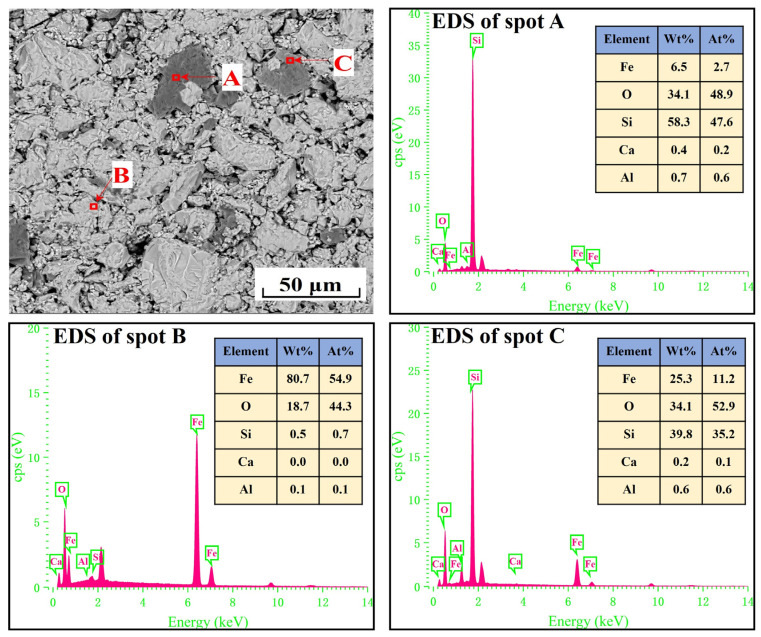
SEM-EDS analysis of iron ore pellets.

Figure 2 shows the scanning electron microscopy and energy-dispersive X-ray spectroscopy (SEM-EDS) analysis of iron ore pellets. They had a relatively compact structure. Moreover, the EDS analysis of spots A, B, and C confirmed the existence of hematite, magnetite, and quartz, agreeing with the result in Figure 1.

### 2.2. Methods

**Figure 3:**
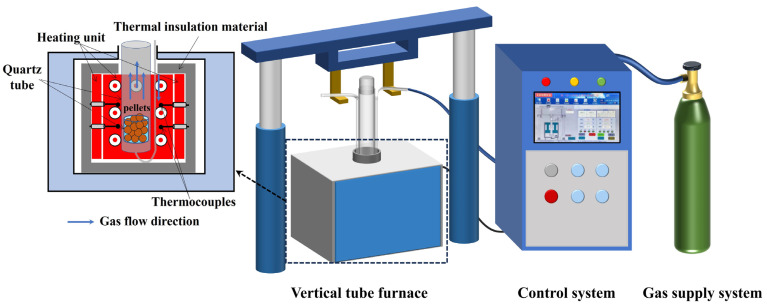
Schematic illustration of experimental setup for reduction.

Before the reduction, 40 g of iron ore pellets were firstly loaded in the quartz tube (ø 40 mm × 510 mm) of a vertical tube furnace (KHGT-30, Kehui Furnace Industry Technology Co., Ltd., Changsha, Hunan, China), as shown in Figure 3. The pellets were then heated to 1150 °C with the heating rate of 10 °C/min in 0.5 L/min N_2_. After keeping the pellets at this temperature for 30 min, the gas was switched to H_2_ with the flow rate of 0.4 L/min to start the reduction process with different cooling rates, i.e., 5.2 °C/min, 4.5 °C/min, 3.7 °C/min, and 2.0 °C/min, respectively, which were determined by considering the variation and control of the rate in the entire HCR process. When the temperature dropped to 450 °C, the gas was switched back to 0.5 L/min N_2_ for protection and cooling to room temperature. During the reduction process, an electronic balance was used to record the mass changes of the pellets.

### 2.3. Characterizations

The total iron contents of the pellets were determined by chemical titration according to the Chinese National Standard Test Method GB/T 6730.5-2022 [23]. The ferrous iron and metallic iron contents of the pellets after reduction were determined to calculate the reduction degree and iron metallization degree according to the Chinese National Standard Test Method GB/T 24236-2009 [24]. The reduction degree was calculated using the following equation:

(1) $$RD=\left\{\frac{0.111W_{1}}{0.430W_{2}}+\frac{m_{0}-m_{t}}{m_{0}\times 0.430W_{2}}\times 100\right\}\times 100\%$$

where W_1_ is the ferrous content of the pellets before reduction, wt%; W_2_ is the total iron content of the pellets before reduction, wt%; m_0_ is the mass of the pellets before reduction, g, and m_t_ is the mass of the pellets after reduction, g. The iron metallization degree (MD) was calculated using the following equation:

(2) $$MD=\frac{MFe}{TFe}\times 100\%$$

where MFe is the metallic iron content of the metallized pellets, wt.%, and TFe is the total iron content of the metallized pellets, wt%. The compressive strength of the pellets was measured according to the Chinese National Standard Test Method GB/T 14201-2018 [25].

The phase compositions of the pellets were determined using an X-ray diffraction spectrometer (D8 Advance, Bruker, Karlsruhe, Germany). The morphologies/microstructures of the metallized pellets, which were polished and embedded in epoxy resin, were characterized using a scanning electron microscope equipped with an energy-dispersive X-ray spectrometer (Quanta 250FEG, FEI, Brno, Czech Republic). The porosities of different areas of the metallized pellets were determined based on the analysis of SEM images of cross-sections of the pellets using the method proposed by Otsu [26,27]. The pore distributions of the metallized pellets, which were embedded in epoxy resin and polished in advance, were determined based on the EDS line scan results of the elements including carbon, iron, and oxygen [20]. The total porosities of the pellets were characterized according to the Chinese National Standard Test Method GB/T 24586-2009 [28]. The iron particle sizes after hydrogen reduction with different cooling rates were calculated by processing the sectioned microscopic images of qualified metallized pellets using the software Image J 2.0 (National Institutes of Health, Bethesda, MD, USA). The Feret diameter, defined as the distance between two boundary lines of the projected contour of an object’s particles measured along any direction, was used to describe the average iron particle size [29,30].

## 3. Results and Discussion

### 3.1. Effect of Cooling Rate on the Reduction Indexes

**Figure 4:**
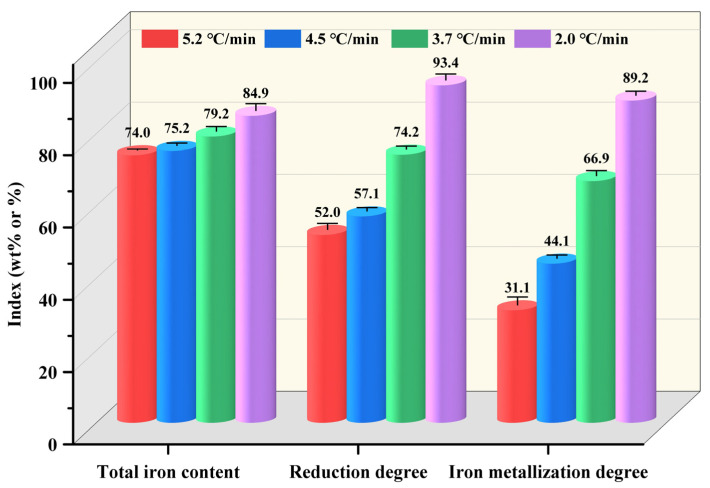
Total iron contents, reduction degrees, and iron metallization degrees of the metallized pellets obtained by reduction with different cooling rates.

To investigate the effect of cooling rate on the HCR process, Figure 4 shows the variations of reduction indexes of the pellets with cooling rate. As the cooling rate declined from 5.2 °C/min to 2.0 °C/min, the total iron content of metallized pellets increased from 74.0 wt% to 84.9 wt%. Meanwhile, the reduction degree and iron metallization degree increased from 52.0% and 31.1% to 93.4% and 89.2%, respectively.

**Figure 5:**
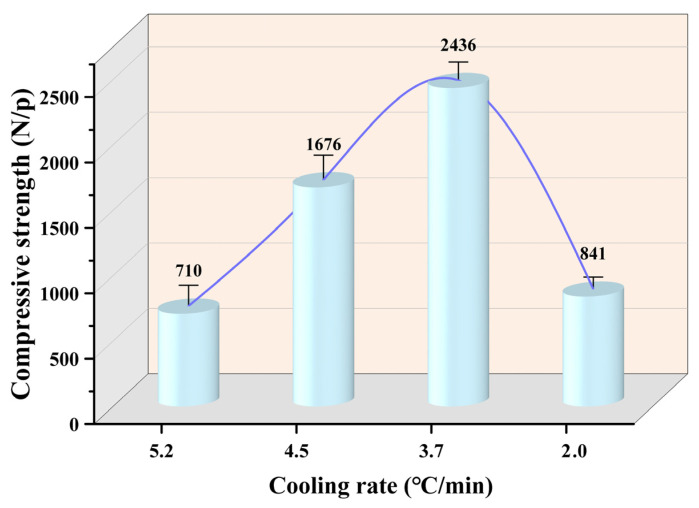
Compressive strength of the metallized pellets obtained by reduction with different cooling rates.

Along with the changes in the above indexes, the compressive strength of the pellets varied evidently. Figure 5 shows the compressive strength of the metallized pellets obtained by HCR with different cooling rates. As shown in Figure 5, when the cooling rate was 5.2 °C/min, the compressive strength of metallized pellets was only 710 N/p, indicating a huge decrease in the strength compared with the iron ore pellets. This was because under the condition of rapid cooling, the internal stress in the pellets could not be released in time, which would induce “cold embrittlement” [31]. As the cooling rate declined, more iron metallization occurred and the compressive strength tended to increase. Under the influence of these two factors, the compressive strength of the pellets reached the maximum, 2436 N/p, when the cooling rate was 3.7 °C/min. However, further decreasing the cooling rate negatively affected the compressive strength. In specific, as the cooling rate was 2.0 °C/min, the compressive strength decreased to only 841 N/p. This phenomenon was mainly associated with the increases in reduction degree and iron metallization degree, which enhanced the plastic deformation capacity of the pellets [32]. It was also partially ascribed to the formation of pores in the reduction process due to various gas–solid reactions which reduced the strength of the pellets [33].

It was reported that the compressive strength of iron ore pellets needs to be higher than 2500 N/p for ironmaking in large blast furnaces [34]. When the cooling rate was 3.7 °C/min, the compressive strength of the metallized pellets basically met this requirement [35]. Therefore, the pellets could be used as a feedstock for the blast furnace ironmaking process. In addition, they could act as an iron-containing coolant in the steelmaking process [36].

**Figure 6:**
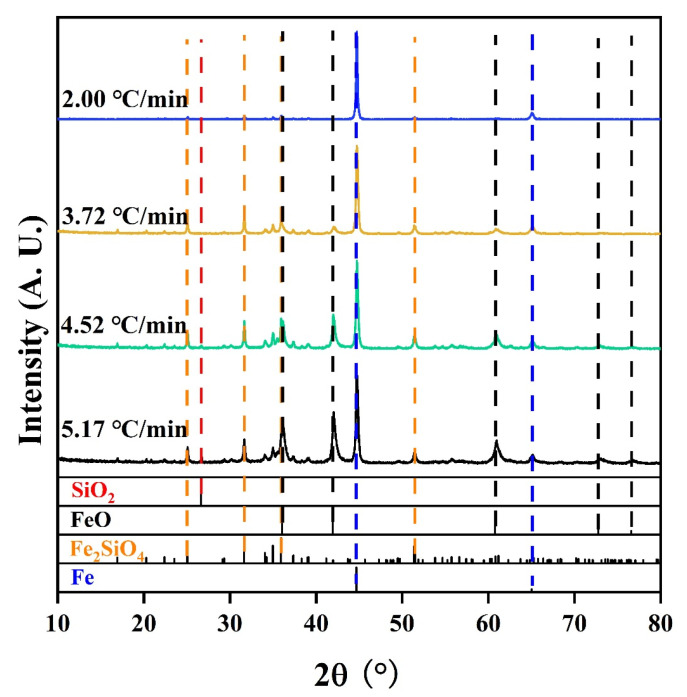
XRD patterns of the metallized pellets obtained by reduction with different cooling rates.

During the reduction process, the phase composition of the pellets was expected to change. Figure 6 shows the XRD patterns of the pellets after HCR with different cooling rates. When the cooling rate was 5.2 °C/min, hematite and magnetite in the pellets were reduced by H_2_ to metallic iron and FeO. Partial trivalent iron was converted to divalent iron which combined with silicon to form Fe_2_SiO_4_, indicating insufficient reduction [37]. As the cooling rate decreased, the diffraction peaks of metallic iron became stronger with declining peaks of FeO and Fe_2_SiO_4_, confirming more complete reduction of the pellets.

**Figure 7:**
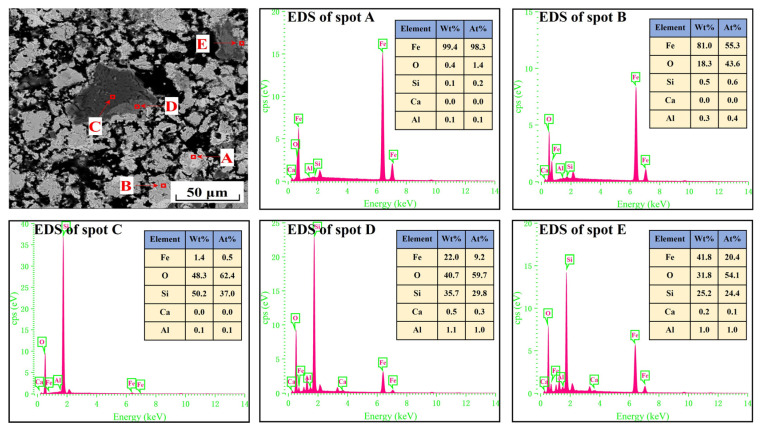
SEM-EDS analysis of the metallized pellets obtained by reduction with the cooling rate of 5.2 °C/min.

**Figure 8:**
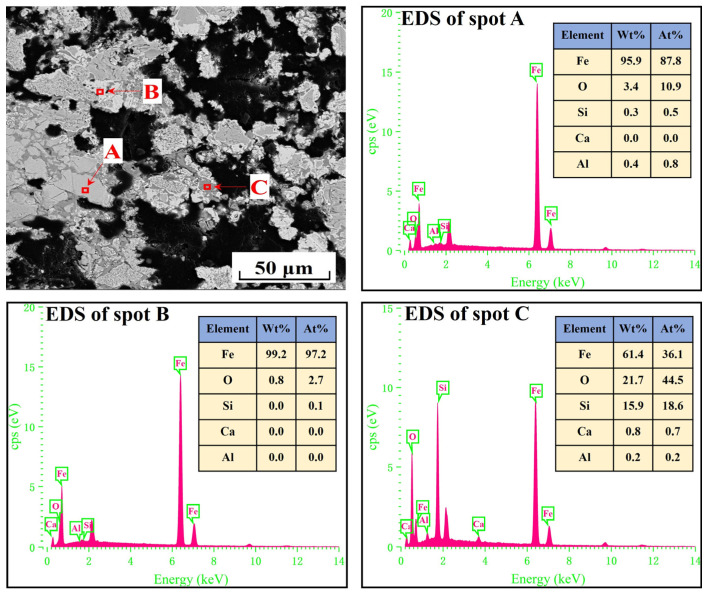
SEM-EDS analysis of the metallized pellets obtained by reduction with the cooling rate of 4.5 °C/min.

**Figure 9:**
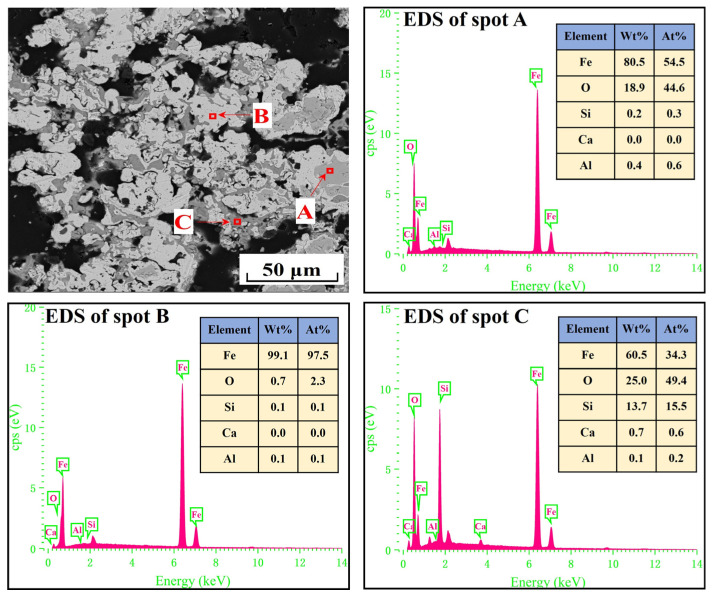
SEM-EDS analysis of the metallized pellets obtained by reduction with the cooling rate of 3.7 °C/min.

**Figure 10:**
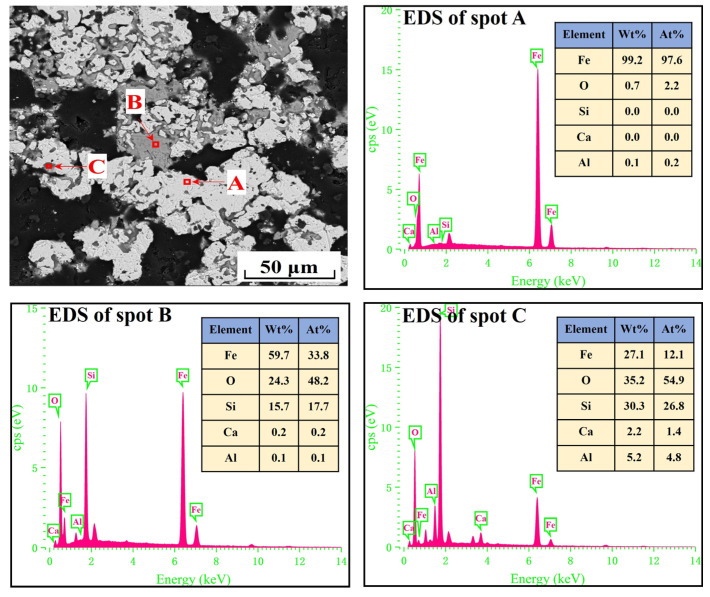
SEM-EDS analysis of the metallized pellets obtained by reduction with the cooling rate of 2.0 °C/min.

For further analysis of the differences of the metallized pellets obtained by reduction with different cooling rates, Figure 7, Figure 8, Figure 9 and Figure 10 show the SEM-EDS analyses of the metallized pellets. After reduction with the cooling rate of 5.2 °C/min, the metallic iron particles were distributed in a fragmented manner, with clear gaps between them. With decreasing cooling rate, the particles tended to aggregate. Moreover, the gaps between the particles were reduced and a certain quantity of pores were generated. It explained the changing trend of compressive strength from the microscopic point of view. When the cooling rate declined to 2.0 °C/min, the metallic iron particles became more aggregated, with large pores between them, which reduced the compressive strength of the metallized pellets.

The EDS analysis of the spots in Figure 7 and Figure 8 revealed that metallic iron particles were preferentially generated from the edges of FeO particles and continuously diffused inward as the cooling rate decreased. Large SiO_2_ particles gradually disappeared and transformed into Fe_2_SiO_4_ existing between the FeO particles. As the cooling rate decreased further, according to the EDS analysis of spots A, B, and C in Figure 9, the nascent iron aggregates kept growing to encapsulate unreacted FeO and Fe_2_SiO_4_. When the cooling rate was 2.0 °C/min, complete reduction was identified, as confirmed by the EDS analysis in Figure 10. In specific, FeO was completely converted to Fe and Fe_2_SiO_4_ was partially converted to Fe and SiO_2_.

**Figure 11:**
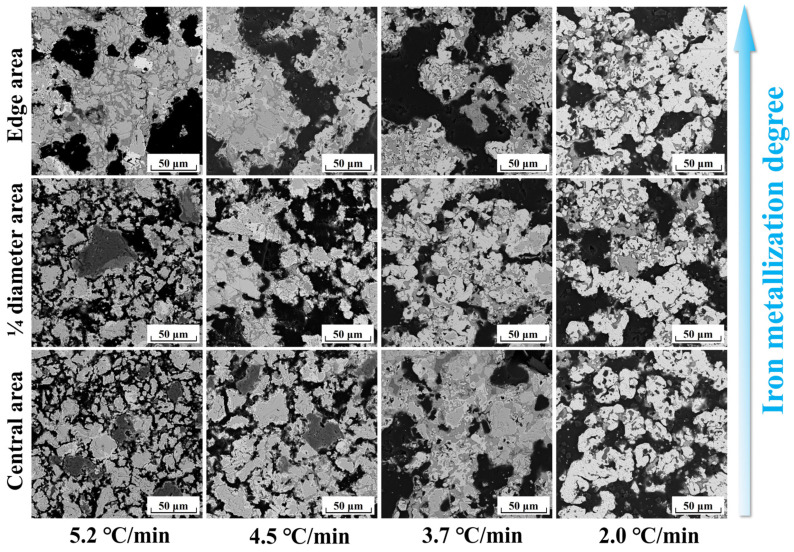
Microstructures of different areas of the metallized pellets obtained by reduction with different cooling rates.

To further examine the extent of reduction in different regions inside the metallized pellets obtained under different conditions, the microstructures of the metallized pellets at their edge, 1/4 diameter, and central areas were characterized, as shown in Figure 11. The metallization degree of the metallized pellets increased from the center to the edge after reduction with different cooling rates. When the cooling rate was 5.2 °C/min, the metallized pellets had obvious metallization at the edges. Only a few metallic iron particles were formed at the 1/4 diameter and at the center. It verified that with a high cooling rate, the temperature of the system decreased rapidly and could not provide sufficient thermal energy for reduction reactions. Decreasing cooling rate significantly promoted the reactions at both the edge and 1/4 diameter area, especially for reduction with the cooling rate of 3.7 °C/min. As the cooling rate was 2.0 °C/min, the difference between these regions almost disappeared.

In addition to the regional differences in metallization degree, the changes in porosity can be clearly observed in Figure 11. There was a similar trend to that of iron metallization degree. In other words, the porosity increased with increasing iron metallization degree. When the cooling rate decreased to 2.0 °C/min, the metallized pellets became more porous, lowering their compressive strength.

### 3.2. Effect of Cooling Rate on the Porosity

**Figure 12:**
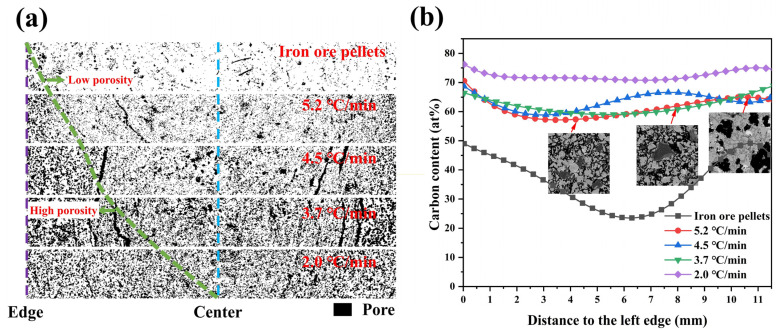
(**a**) Pore distributions and (**b**) fluctuations in carbon content along the radial direction in the metallized pellets obtained by reduction with different cooling rates.

As aforementioned, the porosity varied with the cooling rate during the reduction process. To describe the trend more accurately, the distribution of porosity inside different metallized pellets was analyzed. Figure 12a shows the pore distributions of different pellets from the edge to the center. More pores could be clearly observed in the left side of the green line. The porosity of the metallized pellets increased considerably with decreasing cooling rate. At the same time, this change progressed inward. The interior of the metallized pellets could be divided into the high-porosity region and low-porosity region. By combining with the line-scan fitting curve of elemental carbon from epoxy resin in Figure 12b, it was found that the carbon content of the pellets after reduction increased significantly, indicating the increase in porosity of the pellets via reduction. It was related to the migration and aggregation of metallic iron particles and emission of the reduction product, H_2_O, during the reduction process. Meanwhile, as the reduction process proceeded, the fluctuations in carbon content along the radial direction gradually became smaller, indicating the relatively uniform pore distribution.

**Figure 13:**
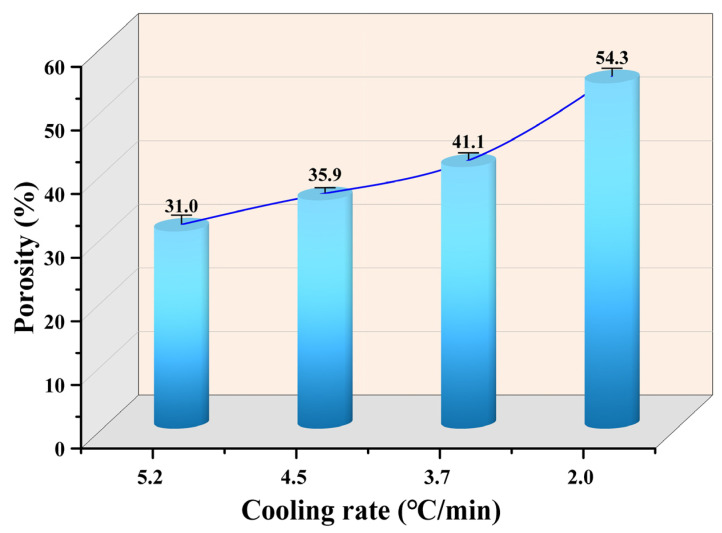
Porosities of the metallized pellets obtained by reduction with different cooling rates.

Figure 13 shows the porosities of the metallized pellets obtained by reduction with different cooling rates. Compared to the iron ore pellets, the porosity of the metallized pellets increased by 29.7%, 50.2%, 72.0%, and 127.2% when the cooling rate was 5.2 °C/min, 4.5 °C/min, 3.7 °C/min, and 2.0 °C/min, respectively. Although a larger porosity indicated a higher reduction degree and iron metallization degree, it was detrimental to the compressive strength. Therefore, selecting a proper cooling rate would be critical for HCR.

### 3.3. Effect of Cooling Rate on the Generation of Metallic Iron Particles

**Figure 14:**
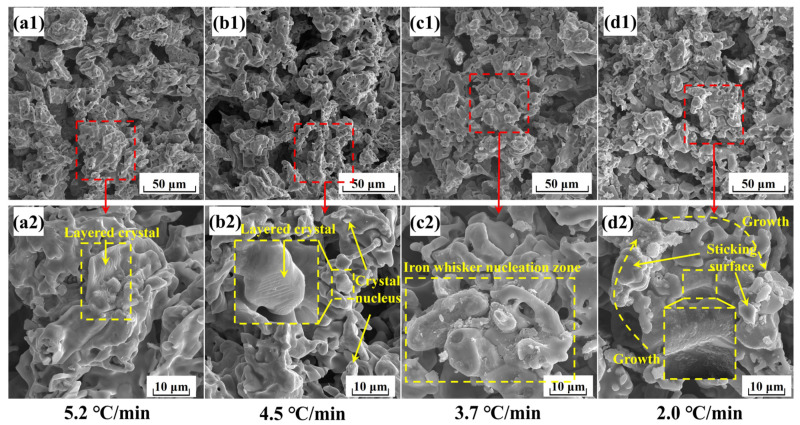
Micro-morphologies of the metallized pellets obtained by reduction with different cooling rates.

In the HCR process, metallic iron particles would form with variable morphological features. Figure 14 shows the micro-morphologies of the metallized pellets obtained by reduction with different cooling rates.

According to Figure 14, when the cooling rate was 5.2 °C/min, there were a number of layered iron particles with relatively smooth surfaces, consistent with the iron precipitation morphology found below 800 °C [38]. It could be attributed to the rapid temperature decrease and the short dwell time at high temperatures.

When the cooling rate decreased to 4.5 °C/min, more nascent iron particles formed on the surfaces of layered iron particles. Because the dwell time of the reaction system in the high temperature range (>900 °C) was extended with this cooling rate, the precipitation rate of metallic iron increased and the mode of precipitation shifted from the steady state to the unsteady state [39]. In this case, both layered and small spherical iron particles co-existed.

Figure 14 also shows the morphologies of the metallized pellets when the cooling rate was 3.7 °C/min. Because of the prolonged dwell time in the high temperature range, most of metallic iron nucleated at points and grew into iron whiskers. At the same time, due to the high reduction potential on the surface of the pellets, the reduction reaction proceeded rapidly and the migration rate of oxygen atoms was larger than the precipitation rate of iron atoms, leading to the precipitation of a small portion of metallic iron in the form of porous reticular structure. With this cooling rate, non-steady state precipitation became dominant.

When the cooling rate decreased to 2.0 °C/min, the iron whiskers on the surfaces of the metallized pellets had longer lengths. Meanwhile, more metallic iron precipitated in the porous reticular form. It microscopically explained the huge increase in porosity and the considerable decrease in compressive strength.

**Figure 15:**
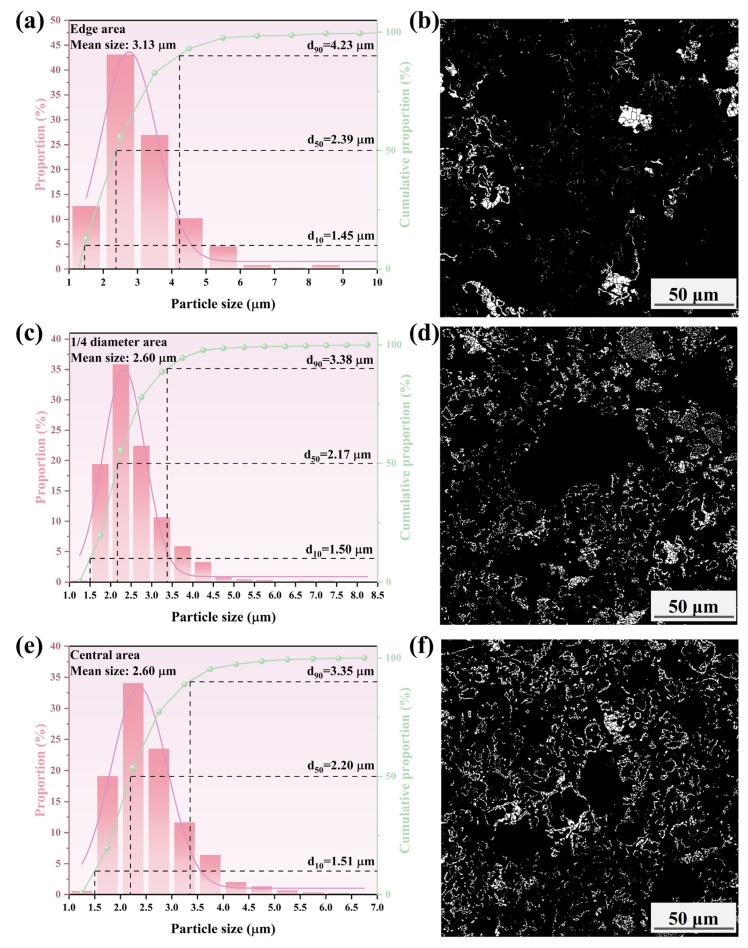
Histograms and microscopic features of iron particle size distributions in different areas of the metallized pellets obtained by reduction with the cooling rate of 5.2 °C/min: (**a**,**b**) edge area, (**c**,**d**) 1/4 diameter area, and (**e**,**f**) central area.

**Figure 16:**
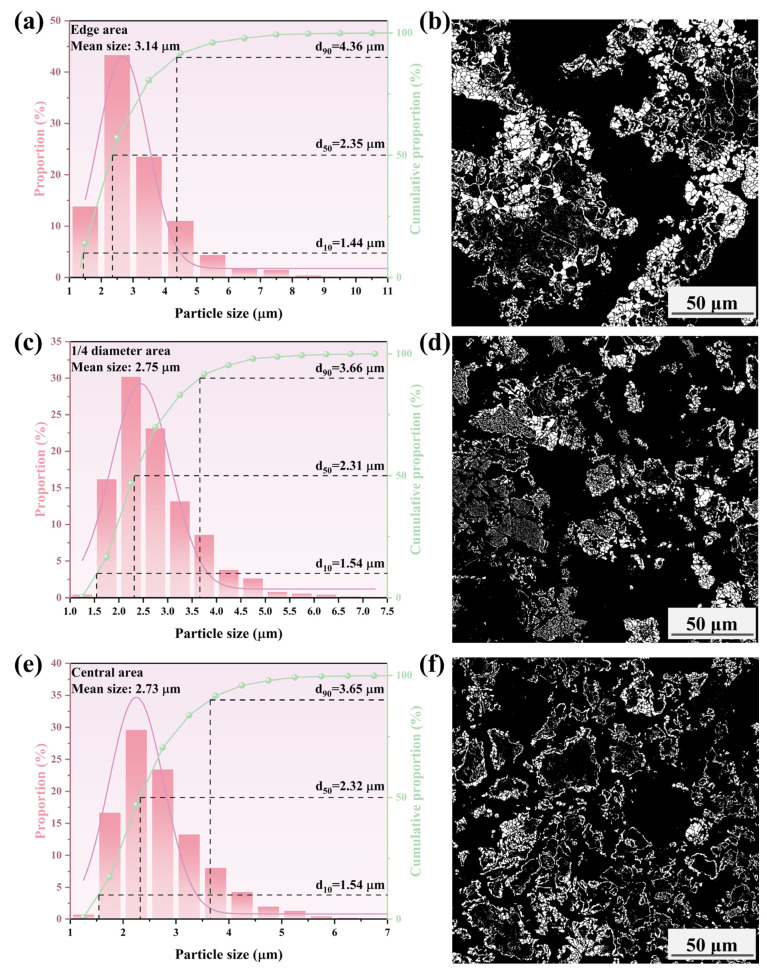
Histograms and microscopic features of iron particle size distributions in different areas of the metallized pellets obtained by reduction with the cooling rate of 4.5 °C/min: (**a**,**b**) edge area, (**c**,**d**) 1/4 diameter area, and (**e**,**f**) central area.

**Figure 17:**
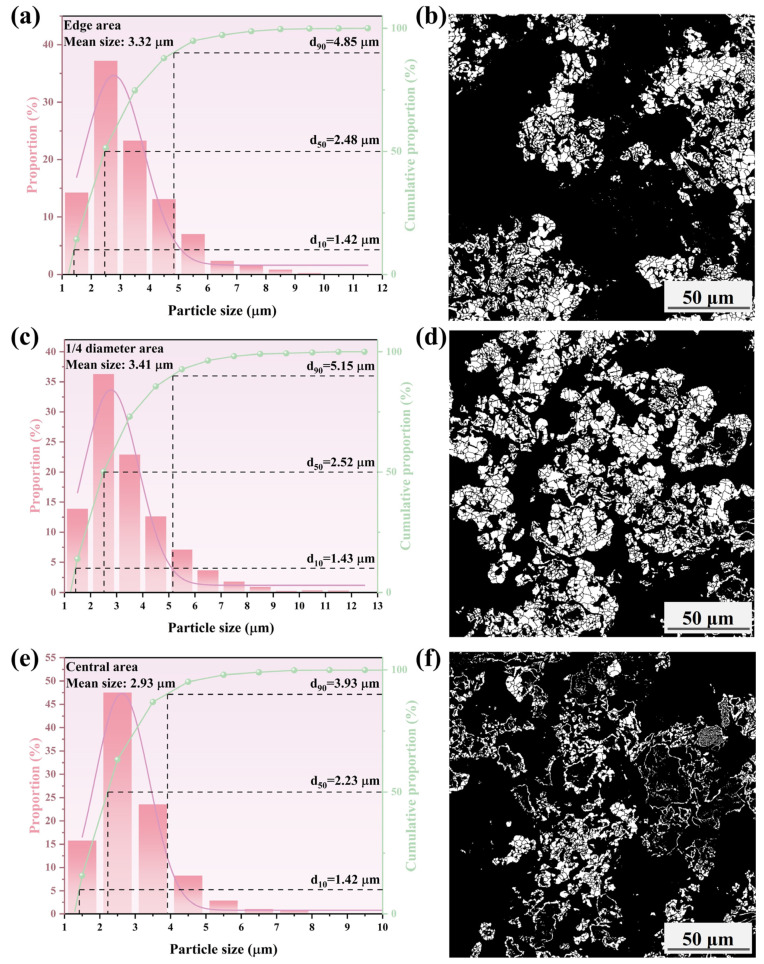
Histograms and microscopic features of iron particle size distributions in different areas of the metallized pellets obtained by reduction with the cooling rate of 3.7 °C/min: (**a**,**b**) edge area, (**c**,**d**) 1/4 diameter area, and (**e**,**f**) central area.

**Figure 18:**
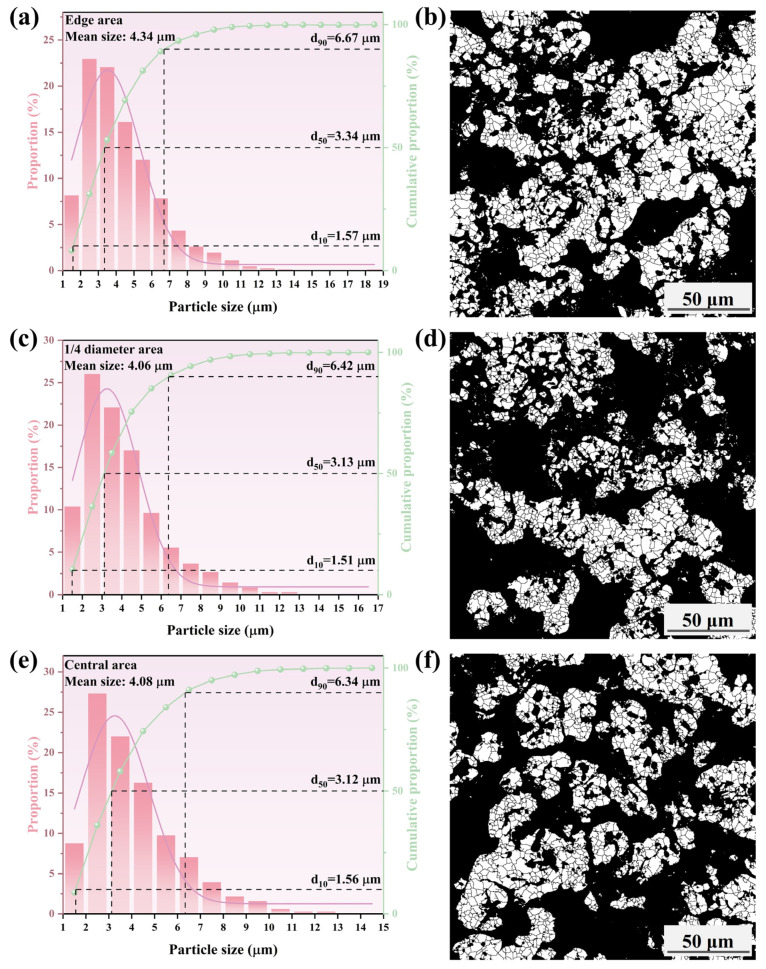
Histograms and microscopic features of iron particle size distribution in different areas of the metallized pellets obtained by reduction with the cooling rate of 2.0 °C/min: (**a**,**b**) edge area, (**c**,**d**) 1/4 diameter area, and (**e**,**f**) central area.

To further assess the effect of the cooling rate on the growth behavior of metallic iron particles, the iron particle sizes in different areas of the metallized pellets were characterized. The results are shown in Figure 15, Figure 16, Figure 17 and Figure 18.

Figure 15, Figure 16, Figure 17 and Figure 18 show that the mean size of metallic iron particles at the edge area of the metallized pellets was larger than those at 1/4 diameter and the central areas of the pellets. This was particularly evident at high cooling rate. Moreover, the d_10_ values of metallic iron particles of different metallized pellets were all around 1.5 μm. However, the d_50_ value of metallic iron particles in the metallized pellets increased significantly with decreasing cooling rate due to longer dwell time in the high temperature range.

As shown in Figure 15, only a small quantity of metallic iron particles larger than 5 μm were observed at the edges. The size distribution of metallic iron particles at 1/4 diameter area of the pellets was approximately the same as that at the central area of the pellets, with more than 90% of the particles being smaller than 3.4 μm. According to Figure 16, when the cooling rate decreased to 4.5 °C/min, the metallic iron particles grew obviously along the edges of FeO particles and spread inward. The mean size increased from 3.13 μm (edge area), 2.60 μm (1/4 diameter area), and 2.60 μm (central area) to 3.14 μm (edge area), 2.75 μm (1/4 diameter area), and 2.73 μm (central area). When the cooling rate decreased to 3.7 °C/min, as shown in Figure 17, the mean sizes of metallic iron particles were 3.32 μm (edge area), 3.41 μm (1/4 diameter area), and 2.93 μm (central area). It indicated that the reduction performance was comparable at the edge and 1/4 diameter areas of the pellets, while the reduction at the central area was relatively incomplete. As expected, further reducing the cooling rate to 2.0 °C/min promoted the reduction of the pellets. As shown in Figure 18, the mean sizes of metallic iron particles reached 4.34 μm (edge area), 4.06 μm (1/4 diameter area), and 4.08 μm (central area). In addition, the distribution range and quantity of metallic iron particles in these three areas were nearly the same. It revealed that the reduction of the pellets became more uniform.

## 4. Conclusions

In this study, the effect of cooling rate on the HCR of iron ore pellets was investigated. When the cooling rate decreased from 5.2 °C/min to 2.0 °C/min, the total iron content, reduction degree, and iron metallization degree of the pellets increased from 74.0 wt%, 52%, and 31.1% to 84.9 wt%, 93.4%, and 89.2%, respectively. However, the compressive strength of the pellets increased initially from 2100 N/p to 2436 N/p and subsequently decreased significantly to 841 N/p. A too fast or too slow cooling rate would negatively affect the compressive strength of the pellets. During the reduction process, the main phase of the pellets, Fe_2_O_3_, had stepwise reactions to Fe, with diminishing FeO and Fe_2_SiO_4_. As the cooling rate declined, the porosity of the pellets increased from 23.9% to 54.3%, with a higher distribution uniformity of pores. The morphology of metallic iron particles transited from a layered form to a spherical form and finally to a porous reticular form. Meanwhile, the metallic iron particles in the pellets grew evidently and distributed more uniformly based on the analysis of metallic iron particle sizes in different areas of the pellets, namely edge area, 1/4 diameter area, and central area. After reduction with the cooling rate of 3.7 °C/min, the resulting metallized pellets had the reduction degree of 74.2%, iron metallization degree of 66.9%, and the highest compressive strength of 2436 N/p, mainly associated with the spherical morphology and relatively large size of metallic iron particles. Further efforts will be made to obtain the balance between iron metallization degree and compressive strength of the pellets.

## Figures and Tables

**Table 1:** Main chemical composition of iron ore pellets (wt%).

TFe	SiO_2_	Al_2_O_3_	CaO	Na_2_O	MgO	K_2_O	P	S	LOI
64.00	8.51	0.42	0.18	0.18	0.15	0.10	0.045	0.0046	2.58

**Table 2:** Physical characteristics of iron ore pellets.

Item	Size (mm)	Compressive Strength (N/p)	True Density (g/cm^3^)	Bulk Density (g/cm^3^)	Porosity (%)
Value	14–16	2100	4.45	3.39	23.9

## Data Availability

The datasets presented in this article are not readily available because the data are part of an ongoing study. Requests to access the datasets should be directed to zwpeng@csu.edu.cn.
